# Correlates of long duration of untreated illness (DUI) in patients with bipolar disorder: results of an observational study

**DOI:** 10.1186/s12991-023-00442-5

**Published:** 2023-03-23

**Authors:** Gabriele Di Salvo, Giorgia Porceddu, Umberto Albert, Giuseppe Maina, Gianluca Rosso

**Affiliations:** 1 San Luigi Gonzaga University Hospital, Department of Neurosciences ‘Rita Levi Montalcini’, University of Turin, Turin, Italy; 2 Department of Neurosciences ‘Rita Levi Montalcini’, University of Turin, Turin, Italy; 3 Department of Medicine, Surgery and Health Sciences, University of Trieste, Trieste, Italy; 4 UCO Clinica Psichiatrica, Azienda Sanitaria Universitaria Giuliano Isontina, ASUGI, Trieste, Italy

## Abstract

**Background:**

Despite a high number of studies investigating the correlation between long Duration of Untreated Illness (DUI) and poor course of Bipolar Disorder (BD), the results concerning the impact of DUI on some specific factors, such as suicidality and medical comorbidities, are still inconsistent. This cross-sectional observational study aimed at analyzing potential socio-demographic and clinical correlates of long DUI in a large cohort of real-world, well-characterized BD patients.

**Methods:**

The socio-demographic and clinical characteristics of 897 patients with BD were collected. The sample was divided for analysis in two groups (short DUI vs long DUI) according to a DUI cutoff of 2 years. Comparisons were performed using χ^2^ tests for categorical variables and the Kruskal–Wallis test for continuous variables. Logistic regression (LogReg) was used to identify explanatory variables associated with DUI (dependent variable).

**Results:**

Six-hundred and sixty patients (75.5%) presented long DUI (> 2 years) and mean DUI was 15.7 years. The LogReg analysis confirmed the association of long DUI with bipolar II disorder (*p*: 0.016), lower age at onset (*p* < 0.001), depressive predominant polarity (*p*: 0.018), depressive polarity onset (*p* < 0.001), longer duration of illness (*p* < 0.001), lifetime suicide attempts (*p*: 0.045) and current medical comorbidities (*p*: 0.019).

**Conclusions:**

The present study confirms the association between long DUI and higher risk of suicide attempts in patients with BD. Moreover, an association between long DUI and higher rates of medical conditions has been found.

## Introduction

Bipolar disorder (BD) is a prevalent and severe psychiatric disease, included among the world’s ten most disabling conditions according to the World Health Organization [1].

Duration of untreated illness (DUI), defined as the time span from disorder onset to proper diagnosis and adequate treatment, has been increasingly investigated in several psychiatric disorders as a possible predictor of illness course specifier [2, 3], such as symptomatic severity, remission, response to treatment and global functioning in psychoses [4], but also in mood disorders, including BD [5].

A large number of studies show that the DUI, is long in BD [6–10]. The reasons for long DUI in BD have been widely addressed by clinical research. First, the onset of BD usually takes place in late childhood/early adulthood [11], a key developmental period which can partly explain delays in diagnosis and treatment, due to possible reluctance in reaching a definitive diagnosis [12]. Moreover, BD is characterized by a complex clinical picture and can be misdiagnosed: manic episodes with psychotic features may fail to be differentiated from psychotic disorders, acute depressive episodes often occur before (hypo)manic symptoms and many patients with ‘soft’ bipolar disorders can easily be misdiagnosed as patients with personality disorders [13–15]. Further barriers standing in the way of BD patients seeking adequate diagnosis and treatment include social stigma and lack of easily accessible treatment centers [13, 16, 49].

Despite a high number of studies investigating the correlation between long DUI and poor course of BD, the results concerning the impact of DUI on some specific factors, such as suicidality and number of lifetime affective episodes, are still inconsistent.

Indeed, some studies showed longer DUI to be associated with higher rates of suicide attempts [5, 6, 16, 17], a higher number of mood episodes [6, 18] and hospitalizations [13],conversely, other reports failed to find an association between long DUI and, respectively, rapid cycling [19] and number of suicide attempts [20, 21]. Likewise, studies analyzing the correlation between longer DUI in BD and detrimental outcomes such as poor response to treatment [22–24] and worse overall functioning [2–4, 12, 21, 22, 30], have yielded conflicting results.

The aim of this study is to contribute to the knowledge of DUI-associated variables by examining a large naturalistic sample of well-characterized BD patients.

## Methods

### Study design and patients

Data derive from an independent cross-sectional observational study aimed at analyzing course characteristics, medical conditions and response to treatments in in- and out-patients with BD. Subjects were recruited from all patients with a principal diagnosis of BD consecutively referred to the Psychiatric Unit of San Luigi Gonzaga University Hospital in Orbassano (University of Turin, Italy), from January 2014 to February 2022.

To be enrolled, patients had to fulfill the following inclusion criteria: (a) main diagnosis of BD type I, II or Not Otherwise Specified (NOS)(DSM-IV-TR, DSM-5) [25, 26],(b) written consent to participate in the study, after being thoroughly informed about aims and study procedures.

The exclusion criteria were: (a) age < 18; (b) concomitant severe, unstable, active degenerative diseases; (c) refusal to consent participating in the study.

The protocol was approved by the local Ethical Committee. All subjects gave a written informed consent to have their clinical data potentially used for research purposes (provided that these data are anonymously treated).

### Assessment and procedures

Certified psychiatrists or residents in psychiatry supervised by senior psychiatrists performed the clinical assessment of patients.

All diagnoses were confirmed by means of the Mini-International Neuropsychiatric Interview (MINI) [27].

At study entry, general socio-demographic information and clinical data were collected for each subject through the administration of a semi-structured interview that we developed and used in regular clinical practice and in previous studies as well [28].

A retrospective life chart ranging from onset of first mood symptoms until study entry was reconstructed for each participant, resulting in a graphical representation of the past longitudinal course of illness.

DUI was defined as the time from the onset of the first affective episode to the start of the first adequate treatment, as in previous studies [6]. Adequate treatment is defined as the prescription of drugs labelled by European Medical Agency (EMA) for acute episode treatment and/or recurrence prevention of bipolar disorder: lithium, anticonvulsants (valproic acid, lamotrigine), atypical antipsychotics (aripiprazole, olanzapine, quetiapine, asenapine).

Furthermore, all subjects received a medical examination, included the assessment of metabolic parameters.

### Statistical analysis

Patients were divided into two groups according to the DUI length (short: ≤ 2 years vs. long: > 2 years): the cutoff was set at 2 years, as available data suggest that early intervention within this time span can yield a substantial improvement in long-term outcomes of BD [17, 19, 42, 52]. The association between DUI and socio-demographic and clinical factors was analyzed in the entire sample and in two sub-samples (patients with BD type I and patients with BD type II).

The normality of data distribution was evaluated using Kolmogorov–Smirnov test (KS). Since the distribution was not normal (KS: 0.145; *p*: < 0.001), comparisons were performed using χ2 tests for categorical variables and Kruskal–Wallis test for continuous variables.

Furthermore, binary logistic regression (LogReg) was performed setting DUI (categorical variable: long DUI vs short DUI) as the dependent variable. Independent variables were gender, type of BD, age at BD onset, bipolar cycle, predominant polarity, onset polarity, duration of illness, lifetime suicide attempts, comorbid current/lifetime medical conditions.

All statistical analyses were performed by SPSS software version 28.0.1

## Results

Nine-hundred and seven patients with BD were asked to participate; ten refused their consent. Among the 897 patients recruited, 23 (2.6%) were excluded from the research due to lack of data about the DUI.

Ultimately, we completed the analysis using 874 subjects. The demographic and clinical characteristics of the total sample are given in Table 1. The sample is representative for the population of patients with BD: 61.0% of the patients were females, the majority of the sample (54.9%) had bipolar II disorder, the mean age at onset of BD was 29.2 ± 12.4 years, the mean duration of illness was 20.4 ± 14.1 years. The mean DUI of the total sample was 15.7 ± 23.5 years; six-hundred and sixty patients (75.5%) showed a long DUI (> 2 years). The demographic and clinical differences between patients with long and short DUI are summarized in Table 1. Subjects with long DUI, compared with patient with short DUI, showed a higher rate of female gender (62.9% vs 55.1%; *p*: 0.044), a higher percentage of bipolar II disorder (60.6% vs 37.4%; *p*: < 0.001), a lower age at onset (28.0 ± 11.2 vs 32.9 ± 15.1; *p*: > 0.001), a lower rate of MDI bipolar cycle (27.2% vs 38.2%; *p*: 0.026) and a higher rate of irregular cycle (53.8% vs 43.0%; *p*: 0.026), a higher rate of depressive predominant polarity (43.1% vs 22.1%; *p*: < 0.001), a higher rate of depressive onset polarity (73.3% vs 37.4%; *p*: < 0.001), a longer duration of illness (22.8 ± 13.4 years vs 13.1 ± 13.6 years; *p*: < 0.001), more lifetime depressive episodes (4.5 ± 4.2 vs 3.6 ± 3.6; *p*: < 0.001), more lifetime affective episodes (9.4 ± 7.0 vs 7.3 ± 6.6; *p*: < 0.001), a higher rate of lifetime suicide attempts (28.5% vs 17.3%; *p*: 0.001), a lower rate of lifetime involuntary admissions (22.4% vs 47.4%; *p*: < 0.001), higher rates of current (55.3% vs 41.8%; *p*: < 0.001) and lifetime (54.9% vs 40.9%; *p*: < 0.001) medical comorbidities.

**Table 1:** Socio-demographic and clinical characteristics of the total sample (*n* = 874) and differences in socio-demographic and clinical characteristics between patients with long DUI (*n* = 660) or short DUI (*n* = 214)

Characteristics	Total sample (*n* = 874)	Long DUI (*n* = 660)	Short DUI (*n* = 214)	*F*/*χ*^2^	*p*
Age at inclusion (years), mean ± *sd*	47.5 ± 18.1	47.9 ± 18.3	46.1 ± 17.3	1.631	0.202
Education (years), mean ± *sd*	13.6 ± 9.2	14.1 ± 10.3	11.8 ± 4.0	10.707	**0.001**
Sex, *n* (%)					
Male	341 (39.0)	245 (37.1)	96 (44.9)	4.067	**0.044**
Female	533 (61.0)	415 (62.9)	118 (55.1)		
Mood state, type, *n* (%)					
Euthymic	144 (16.5)	113 (17.1)	31 (14.5)	27.494	**< 0.001**
Depressive	489 (55.9)	387 (58.6)	102 (47.7)		
Hypomanic	70 (8.0)	56 (8.5)	14 (6.5)		
Manic	171 (19.6)	104 (15.8)	67 (31.3)		
Bipolar disorder, type, *n* (%)					
Bipolar I	370 (42.3)	242 (36.7)	128 (59.8)	46.927	**< 0.001**
Bipolar II	480 (54.9)	400 (60.6)	80 (37.4)		
Bipolar NOS	24 (2.8)	18 (2.8)	6 (2.8)		
Bipolar cycle, type, *n* (%)					
MDI	261(29.9)	179 (27.2)	82 (38.3)	11.044	**0.026**
DMI	134 (15.4)	101 (15.3)	33 (15.4)		
Irregular	446 (51.1)	354 (53.8)	92 (43.0)		
Rapid cycling	13 (1.5)	11 (1.7)	2 (0.9)		
Continuous	18 (2.1)	13 (2.0)	5 (2.3)		
Age of onset (years), mean ± *sd*	29.2 ± 12.4	28.0 ± 11.2	32.9 ± 15.1	26.044	** < 0.001**
Duration of illness (years), mean ± *sd*	20.4 ± 14.1	22.8 ± 13.4	13.1 ± 13.6	84.514	** < 0.001**
Duration of untreated illness (years), mean ± *sd*	15.7 ± 23.5	20.5 ± 25.1	0.6 ± 0.8	134.19	** < 0.001**
Manic episodes (number), mean ± *sd*	1.3 ± 2.5	1.1 ± 2.2	2.1 ± 2.9	30.381	** < 0.001**
Hypomanic episodes (number), mean ± *sd*	2.5 ± 3.5	2.9 ± 3.5	1.6 ± 2.8	24.623	** < 0.001**
Depressive episodes (number), mean ± *sd*	5.0 ± 4.2	5.4 ± 4.2	3.6 ± 3.6	31.134	** < 0.001**
Affective episodes, total (number), mean ± *sd*	8.9 ± 7.0	9.4 ± 7.0	7.3 ± 6.6	14.772	** < 0.001**
Predominant polarity, type, *n* (%)					
Manic/hypomanic	104 (11.9)	59 (9.0)	45 (21.1)	41.276	** < 0.001**
Depressive	331 (38.0)	284 (43.1)	47 (22.1)		
Onset polarity, type, *n* (%)					
Manic/hypomanic	283 (32.5)	158 (24.0)	125 (58.4)	92.863	** < 0.001**
Depressive	562 (64.4)	482 (73.3)	80 (37.4)		
Lifetime suicide attempts, *n* (%)	225 (25.7)	188 (28.5)	37 (17.3)	10.595	**0.001**
Lifetime involuntary admissions, *n* (%)	116 (28.4)	70 (22.4)	46 (47.4)	22.738	** < 0.001**
Lifetime psychiatric comorbidities, *n* (%)	364 (43.2)	268 (42.7)	96 (44.9)	0.310	0.577
Family history of mood disorders, *n* (%)	505 (57.8)	388 (58.9)	117 (54.7)	1.171	0.279
Current medical comorbidity, *n* (%)	430 (52.1)	348 (55.3)	82 (41.8)	10.896	**0.001**
Lifetime medical comorbidity, *n* (%)	422 (51.6)	343 (54.9)	79 (40.9)	11.486	**0.001**
Weight (kg), mean ± *sd*	72.1 ± 16.3	72.6 ± 16.1	70.8 ± 16.6	1.663	0.198
BMI (kg/m^2^), mean ± *sd*	31.2 ± 98.0	30.0 ± 82.8	34.8 ± 134.3	0.322	0.571
Waist circumference (cm), mean ± *sd*	92.9 ± 17.3	93.5 ± 18.0	90.4 ± 14.2	2.811	0.094
Serum lipid levels (mg/dl), mean ± *sd*					
Tryglicerides	132.4 ± 75.9	131.3 ± 71.8	136.5 ± 89.4	0.565	0.452
HDL cholesterol	52.0 ± 16.4	52.7 ± 16.5	49.3 ± 15.9	4.956	**0.026**
Glycemia (mg/dl), mean ± *sd*	86.7 ± 23.0	86.7 ± 21.7	86.6 ± 26.7	0.002	0.964
Systolic arterial pressure (mmHg), mean ± *sd*	122.6 ± 12.2	122.7 ± 12.3	122.3 ± 11.7	0.152	0.697
Diastolic arterial pressure (mmHg), mean ± *sd*	79.0 ± 8.7	79.2 ± 8.7	78.3 ± 8.6	1.209	0.272
Metabolic syndrome, *n* (%)	235 (32.6)	186 (33.0)	49 (31.0)	0.230	0.631
Abdominal obesity, *n* (%)	265 (48.1)	220 (50.1)	45 (40.2)	3.528	0.060
Low HDL cholesterol, *n* (%)	284 (41.0)	216 (39.6)	68 (46.3)	2.100	0.147
Elevated blood pressure, *n* (%)	352 (50.1)	283 (51.6)	69 (44.5)	2.454	0.117
Impaired fasting glucose, *n* (%)	124 (17.5)	100 (18.0)	24 (15.7)	0.451	0.502
Hypertriglyceridemia, *n* (%)	208 (29.8)	160 (29.2)	48 (31.8)	0.380	0.537

*NOS* not otherwise specified, *BMI* body mass index

The statistically significant results (p<0.005) are in bold type

The LogReg analysis (Table 2) confirmed long DUI to be significantly associated with bipolar II disorder (0.016), lower age at onset (*p*: < 0.001), depressive predominant polarity (*p*: 0.018), depressive polarity onset (*p*: < 0.001), longer duration of illness (*p*: < 0.001), lifetime suicide attempts (*p*: 0.045) and current medical comorbidity (*p*: 0.019).

**Table 2:** Relationship between potential explanatory variables and long DUI: results from the logistic regression analysis

Dependent variables	B	S.E	Wald	*p*
Gender (Male)	0.180	0.184	0.952	0.329
Bipolar disorder, type	0.334	0.138	5.843	**0.016**
Age of onset	−0.42	0.008	29.592	** < 0.001**
Bipolar cycle, type	0.087	0.097	0.817	0.366
Predominant polarity	0.269	0.113	5.626	**0.018**
Onset polarity	1.103	0.190	33.873	** < 0.001**
Duration of illness	1.056	0.135	31.784	** < 0.001**
Lifetime suicide attempts	0.448	0.229	3.827	**0.045**
Current medical comorbidity	0.538	0.229	5.516	**0.019**
Lifetime medical comorbidity	0.372	0.226	2.717	0.099

The statistically significant results (p<0.005) are in bold type

Furthermore, the correlations between DUI and patients characteristics were analyzed in the subgroups of patients with BD type I (n: 370) and patients with BD type II (n: 480).

In the sub-sample of patients with BD I (Table 3), subjects with long DUI, compared with patient with short DUI, showed a higher rate of female gender (64.9% vs 53.1%; *p*: 0.028), a lower age at onset (25.9 ± 13.2 vs 29.4 ± 9.9; *p*: 0.005), a higher rate of depressive predominant polarity (30.7% vs 11.8%; *p*: < 0.001), a higher rate of depressive onset polarity (58.1% vs 24.2%; *p*: < 0.001), a longer duration of illness (23.3 ± 13.6 years vs 15.0 ± 14.1 years; *p*: < 0.001), more lifetime depressive episodes (4.7 ± 3.4 vs 3.4 ± 3.1; *p*: < 0.001), more lifetime affective episodes (8.9 ± 6.0 vs 7.7 ± 5.9; *p*: 0.046), a higher rate of lifetime suicide attempts (25.6% vs 14.1%; *p*: 0.010), a lower rate of lifetime involuntary admissions (46.6% vs 61.1%; *p*: 0.047), higher rates of current (55.3% vs 36.7%; *p*: < 0.001) and lifetime (50.2% vs 38.1%; *p*: 0.032) medical comorbidities.

**Table 3:** Socio-demographic and clinical differences of the BD I sample (*n* = 370) between patients with long DUI (*n* = 242) or short DUI (*n* = 128)

Characteristics	Long DUI (*n* = 242)	Short DUI (*n* = 128)	F/χ^2^	p
Sex, *n* (%)				
Male	85 (35.1)	60 (46.9)	4.851	**0.028**
Female	157 (64.9)	68 (53.1)		
Age at onset (years), mean ± *sd*	25.09 ± 13.2	29.4 ± 9.9	8.150	**0.005**
Predominant polarity, type, *n* (%)				
Depressive	74 (30.7)	15 (11.8)	18.377	** < 0.001**
Onset polarity, type, *n* (%)				
Depressive	140 (58.1)	31 (24.2)	38.623	** < 0.001**
Duration of illness (years), mean ± *sd*	23.3 ± 13.6	15.0 ± 14.1	30.505	** < 0.001**
Depressive episodes (number), mean ± *sd*	4.7 ± 3.4	3.4 ± 3.1	11.022	** < 0.001**
Affective episodes, total (number), mean ± *sd*	8.9 ± 6.0	7.7 ± 5.9	4.033	**0.046**
Lifetime suicide attempts, *n* (%)	62 (25.6)	18 (14.1)	6.599	**0.010**
Lifetime involuntary admissions, *n* (%)	62 (46.6)	44 (61.1)	3.930	**0.047**
Current medical comorbidity, *n* (%)	131 (55.3)	44 (36.7)	11.037	** < 0.001**
Lifetime medical comorbidity, *n* (%)	118 (50.2)	45 (38.1)	4.610	**0.032**

The statistically significant results (p<0.005) are in bold type

In the sub-sample of patients with BD II (Table 4), subjects with long DUI showed a lower age at onset (29.0 ± 11.6 vs 38.0 ± 16.5; *p*: < 0.001), a higher rate of depressive predominant polarity (66.8% vs 51.2%; *p*: 0.03), a higher rate of depressive onset polarity (81.5% vs 60.0%; *p*: < 0.001), a longer duration of illness (22.7 ± 13.3 years vs 10.8 ± 12.6 years; *p*: < 0.001), more lifetime depressive episodes (5.9 ± 4.6 vs 4.1 ± 4.2; *p*: 0.001) and more lifetime affective episodes (9.8 ± 7.6 vs 7.0 ± 7.8; *p*: 0.003).

**Table 4:** Socio-demographic and clinical differences of the BD II sample (*n* = 480) between patients with long DUI (*n* = 400) or short DUI (*n* = 80)

Characteristics	Long DUI (*n* = 400)	Short DUI (*n* = 80)	F/χ^2^	*p*
Age at onset (years), mean ± *sd*	29.0 ± 11.6	38.0 ± 16.5	34.466	** < 0.001**
Predominant polarity, type, *n* (%)				
Depressive	267 (66.8)	41 (51.2)	7.038	**0.030**
Onset polarity, type, *n* (%)				
Depressive	325 (81.5)	48 (60.0)	19.044	** < 0.001**
Duration of illness (years), mean ± *sd*	22.7 ± 13.3	10.8 ± 12.6	54.660	** < 0.001**
Depressive episodes (number), mean ± *sd*	5.9 ± 4.6	4.1 ± 4.2	10.718	**0.001**
Affective episodes, total (number), mean ± *sd*	9.8 ± 7.6	7.0 ± 7.8	8.875	**0.003**

The statistically significant results (p<0.005) are in bold type

## Discussion

The present study aimed at investigating potential socio-demographic and clinical correlates of long DUI in a large cohort of real-world, well-characterized BD patients.

We found a mean DUI of 15.7 years: this result is in line with a previous study conducted by our research group (14.1 years) [29], while other authors report lower DUI in BD [5, 6, 16, 17, 20, 21]. A possible reason for the lack of correspondence between our results and available data is that our Psychiatric Unit is located in a tertiary referral center within the University General Hospital and, in addition, is specialized in the treatment of Mood Disorders: patients suffering from high complexity mood disorders, and thus more likely to receive delayed diagnosis and/or inadequate treatments, are often referred from other centers for a consultation visit. A further possible explanation is that the majority of previous studies [3, 6, 7, 20], unlike ours, enrolled samples mostly represented by BDI patients, more likely presenting a manic onset and, therefore, showing a shorter latency between diagnosis and the prescription of mood-stabilizers as compared with BDII [30],conversely, the predominance of BDII patients in our sample may account for the longer DUI we found. Given the heterogeneity of our sample and the high number of patients with BDII, after carrying out a binary logistic regression (LogReg) in the total sample we also performed separate analyses on the two subsamples of patients with BDI and with BDII, to detect the potential confounding effect of BDII, especially on depressive variables (e.g., depressive onset polarity, depressive predominant polarity).

Concerning socio-demographic variables, our analysis found longer DUI in women: nevertheless this correlation, shown by the Kruskal–Wallis test to be significant, was not confirmed by LogReg analysis and, as already hypothesized by Drancourt and colleagues (2013), is probably to be attributed to clinical confounders, such as bipolar II type and depressive predominant polarity.

Age at onset proved to be significantly lower in the long DUI subsample, in line with previous studies [6–9, 13, 16, 17, 31]. The failure to diagnose BD in young patients can be explained by the highly polymorphous and complex clinical presentation of early onset cases [32], but it may also reflect a lack of awareness of the peak age of BD onset or a reluctance to make a diagnosis with lifetime implications and persistent social stigma [33]. This finding can also account for the longer duration of illness detected in the subgroup of patients with long DUI.

Our analysis found bipolar II subtype to be related to a longer DUI, in accordance with previous studies [3, 5, 6]. This was an expected result, because BD II frequently has a depressive onset [34], that can hamper BD diagnosis and consequently cause delays in prescribing the appropriate treatment. Moreover, hypomania is rarely reported spontaneously [35, 36], and even clinicians often fail to properly recognize it or to consider the clinical implications of such a presentation [37].

Unsurprisingly, our data showed in the subgroup of patients with long DUI higher rates of depressive predominant polarity course and of depressive polarity onset; these results are consistent with previous literature [5–7, 16, 17, 38] and were significant both in the BDI and BDII subgroups. A depressive onset and a subsequent depressive predominant polarity need to be carefully addressed in clinical practice. To improve the diagnosis of BD and thus shorten the DUI, special attention must be paid to some potential predictors of bipolarity, such as abrupt onset, post-partum episodes, treatment resistance and family history of BD or suicide [39],using specific screening tools designed to detect hypomanic symptoms, such as the Hypomania Check List (HCL-32), can also represent a valid aid [40–42].

An interesting result found by our study is the correlation between longer DUI and a higher rate of lifetime suicide attempts. The relationship between suicide risk and DUI is worthy of interest and deserves to be explored by further methodologically rigorous studies, given the crucial clinical implications and the inconsistency of available literature [5, 6, 16, 17, 20, 21]. Our result, if confirmed, could potentially contribute to improve the understanding of suicide attempts and suicide in people with BD and may be helpful in identifying specific targets for suicide prevention interventions, which are still one of the most difficult challenges in daily clinical practice [43]. In addition, our finding may offer indirect support to studies proposing that the introduction of mood stabilizers provides protection against attempted and completed suicide [44, 45], especially lithium because of its proven anti-suicidal effect [46].

A further remarkable result of this study is the significant correlation between longer DUI and a higher rate of concurrent medical comorbidities. This finding confirms, on a larger sample, findings from a previous study performed by our research group on an independent sample [29],to our knowledge, there are no other studies highlighting the relationship between DUI and medical conditions associated with BD. This reason behind this link could be that patients who have been undertreated for years have the tendency to adopt unhealthy lifestyles and often have limited access to care. In addition, patients not undergoing proper treatment could experience higher levels of stress, which could raise cortisol levels, increasing the risk of developing poor glucose tolerance, diabetes, and hypertension. Our results highlight the importance of a thorough assessment of medical conditions when dealing with BD: potentially severe medical conditions can be prevented by better integrating medical and psychiatric care, thus possibly increasing life expectancy for patients with BD [47].

The results of the study should be interpreted in light of several limitations. First, due to the cross-sectional design, many variables were collected retrospectively, making some data less accurate than in controlled studies. Furthermore, in our research, we analyzed lifetime suicide attempts, but the sample does not include completed suicides, meaning that we are unable to test whether the results are generalizable to suicide deaths; in addition, the period in which suicide attempts occurred was not reconstructed, making it difficult to evaluate the direct relationship with DUI. Moreover, in our study the impact of pharmacological treatment on BD course was not evaluated, due to the lack of data on medication (e.g., type of medication, treatment duration, response to treatment) prior to study entry. Another limitation concerns the heterogeneity of recruited patients in terms of clinical characteristics (e.g., medical conditions, pharmacological treatments), with potential confounding factors.

On the other side, our study has some strengths, mainly the large sample size and the thorough clinical characterization: available studies on DUI in BD enrolled relatively small samples or analyzed patients enrolled and treated in different Regions of Italy, with different psychiatric organizations and potential influences on DUI and relative outcomes [5].

## Conclusions

In conclusion, the present study confirms the association between long DUI and some clinical features of BD, such as bipolar II subtype, early age at onset, depressive predominant polarity and depressive polarity onset. The most remarkable finding is the correlation between longer DUI and two detrimental long-term outcomes in BD, such as suicide attempts and medical comorbidity: as abovementioned, this result may potentially contribute to implement early and tailored interventions for BD patients. Prospective studies examining the long-term clinical outcome of DUI in BD are warranted, to better clarify the causal associations between DUI and BD characteristics and course.

## Data Availability

The data sets used and/or analysed during the current study are available from the corresponding author on reasonable request.

## Abbreviations

BD: Bipolar disorder
DUI: Duration of untreated illness
NOS: Not Otherwise Specified
MINI: Mini-International Neuropsychiatric Interview
EMA: European Medical Agency
BMI: Body mass index
IDF: International Diabetes Federation
SD: Standard deviations
KS: Kolmogorov–Smirnov test
LogReg: Logistic regression
HCL-32: Hypomania Check List
